# Performance Analysis of a HT-PEMFC System with 6FPBI Membranes Doped with Cross-Linkable Polymeric Ionic Liquid

**DOI:** 10.3390/ijms23179618

**Published:** 2022-08-25

**Authors:** Yanju Li, Wei Shao, Zheshu Ma, Meng Zheng, Hanlin Song

**Affiliations:** College of Automobile and Traffic Engineering, Nanjing Forestry University, Nanjing 210037, China

**Keywords:** high-temperature proton-exchange membrane fuel cell, polybenzimidazole, ionic liquid, thermodynamic modeling, parameter study

## Abstract

In this paper, a high-temperature proton-exchange membrane fuel cell (HT-PEMFC) system using fluorine-containing polybenzimidazole (6FPBI) composite membranes doped with cross-linkable polymer ionic liquid (cPIL) is developed and studied. The reliability of the model is verified by a comparison with the experimental data. The performance of the HT-PEMFC system using 6FPBI membranes with different levels of cPIL is analyzed. The results show that when the HT-PEMFC uses 6FPBI membranes with a cPIL content of 20 wt % (6FPBI-cPIL 20 membranes), the single cell power density is 4952.3 $W\cdot m^{-2}$. The excessive cPIL content will lead to HT-PEMFC performance degradation. The HT-PEMFC system using the 6FPBI-cPIL 20 membranes shows a higher performance, even at higher temperatures and pressures, than the systems using 6FPBI membranes. In addition, the parametric study results suggest that the HT-PEMFC system should be operated at a higher inlet temperature and hydrogen pressure to increase system output power and efficiency. The oxygen inlet pressure should be reduced to decrease the power consumption of the ancillary equipment and improve system efficiency. The proposed model can provide a prediction for the performance of HT-PEMFC systems with the application of phosphoric-acid-doped polybenzimidazole (PA-PBI) membranes.

## 1. Introduction

With the depletion of fossil fuels and environmental deterioration, worldwide attention has increasingly focused on developing an energy efficient and eco-friendly power generation system [1,2,3,4,5,6]. Unlike conventional power generation systems [7,8,9], fuel cells are not bound by the Carnot cycle which results in a higher energy-conversion efficiency [10,11,12]. Among different kinds of fuel cells, proton-exchange membrane fuel cells (PEMFCs) are more suitable for future portable, mobile, and stationary applications because of their better durability and faster start-up time [13]. Compared with low-temperature proton-exchange membrane fuel cells (LT-PEMFCs), using conventional Nafion as the membrane, HT-PEMFC based on a Polybenzimidazole (PBI) membrane operating at 120–200 °C has a higher CO tolerance and higher quality waste heat [14]. In addition, the HT-PEMFC accelerates the reaction kinetics at the electrodes and simplifies water and heat management [15].

PBI is an amorphous, rigid polymer with good chemical resistance and mechanical strength [16]. PBI is usually doped with phosphoric acid (PA), which is a good electrolyte with low vapor pressure and high thermal stability at high temperatures compared with other acids [17,18,19]. Das et al. [20] reported the first example of the synthesis of porous poly(2,5-benzimidazole) (ABPBI) membranes for HT-PEMFCs using the solvent evaporation/salt leaching techniques. Melchior et al. [21] explained the reason for the better proton conductivity of PBI-phosphoric acid membranes in HT-PEMFCs than in other phosphate-containing electrolytes. Moreover, Asensio et al. [22] and Yu et al. [23] reported that PA was pyrolyzed at 190 °C, resulting in a loss of proton conductivity. The electrical conductivity of PA-PBI polymers depended on the amount of phosphoric acid doped in the polymer [24]. However, when the phosphoric acid content was too high, this led to the degradation of the mechanical properties and a decrease in the electrical conductivity of PA-PBI [25]. Therefore, PBI membranes doped with the appropriate phosphoric acid content helps to improve the output performance of HT-PEMFCs.

The chemical structure of ionic liquids (ILs) containing proton donors and acceptors improved the conductivity of PBI monomers, even at low PA concentrations [26,27,28]. Rajabi et al. [29] used 1,3-di(3-methylimidazole) propane dibromo IL as a dopant for PA-PBI membranes. The results showed that the compounds had enhanced thermal stability, glass transition temperature, and proton conductivity. Compañ et al. [30] combined a series of PA-PBI membranes with different exchangeable anions in ILs to evaluate the effect of the anion and temperature on the proton conductivity of phosphate-doped PBI membranes. In this study, 1-butyl-3-methylimidazole (BMIM) was used as an ionic liquid compound to prepare composite membranes by a casting method with a 5% weight percentage of IL. Liu et al. [31] prepared a series of highly conductive composite membranes based on 6FPBI and poly ionic liquid (PIL). The obtained composite membranes showed enhanced phosphate stability and proton conductivity. A strong correlation between PIL content and proton conductivity was found. In addition, Liu et al. [32] successfully prepared a novel cross-linked composite membrane based on 6FPBI and cPILs for HT-PEMFC applications. The 6FPBI-cPIL membrane achieved extremely high levels of phosphate doping to achieve appreciable proton conductivity. For example, the 6FPBI-cPIL 20 membranes with a PA doping level of 27.8 exhibited a proton conductivity of 0.106${\;S\;\mathrm{cm}}^{-1}$ at 170 °C, which was much higher than that of the pristine 6FPBI membrane. However, researchers have not studied the performance of HT-PEMFC using the 6FPBI-cPIL membrane. Based on the new composite cross-linked membranes, thermodynamic modeling of the HT-PEMFC system using the 6FPBI-cPIL membrane can be developed to predict its performance.

At present, there have been many studies on modeling and parametric studies on PEMFCs. Qin et al. [33] developed a PEMFC stack model based on the flow network method considering the temperature distribution and validated the model using the experimental data. The flow channel was optimized for lower power consumption based on the established PEMFC stack model to obtain the optimal fuel cell stack design. Jo et al. [34] developed and validated a 5-kW HT-PEMFC system model. The reaction kinetics of the fuel conversion process was considered in the model developed for better obtaining the exhaust gas composition and reactor temperature for various operating conditions. The study showed that HT-PEMFC systems required a high degree of thermal integration and optimization of system configuration and operating conditions. A one-dimensional model of an HT-PEM with a PA-doped PBI membrane was developed by Kim et al. [35]. The simulation results indicated that the durability of HT-PEMFCs vary with the current density and PA doping level. Ye et al. [36] discussed the performance of HT-PEMFCs under different operating conditions. The results showed that a higher operating temperature was beneficial to improving the output power and efficiency of the system. However, the relative humidity and operating pressure were not significant to the system performance.

According to that mentioned above, most research has focused on the preparation of ionic membranes and the modeling of fuel cells, while few studies have been conducted on the prediction of HT-PEMFC performance with the application of advanced ionic membranes. In this study, we developed a thermodynamic model of the HT-PEMFC system using 6FPBI-cPIL membranes. The proposed system used the waste heat generated by the stack to preheat the inlet gas to improve the system efficiency. The performance of HT-PEMFCs with different membranes and different operating conditions was analyzed to provide a reference for future fuel cell system design and operation. The following parts of this paper are organized as: Section 2 provides the numerical simulation for membrane applications and the corresponding discussion. The analysis of 6FPBI-cPIL membranes and a model of the HT-PEMFC system based on PA-PBI membranes is established are given in Section 3. The conclusions are presented in Section 4.

## 2. Results and Discussion

The design parameters of the HT-PEMFC single cell can be found in [37]. The input data for the thermodynamic modeling of the system are given in Table 1.

### 2.1. Model Validation

**Figure 1:**
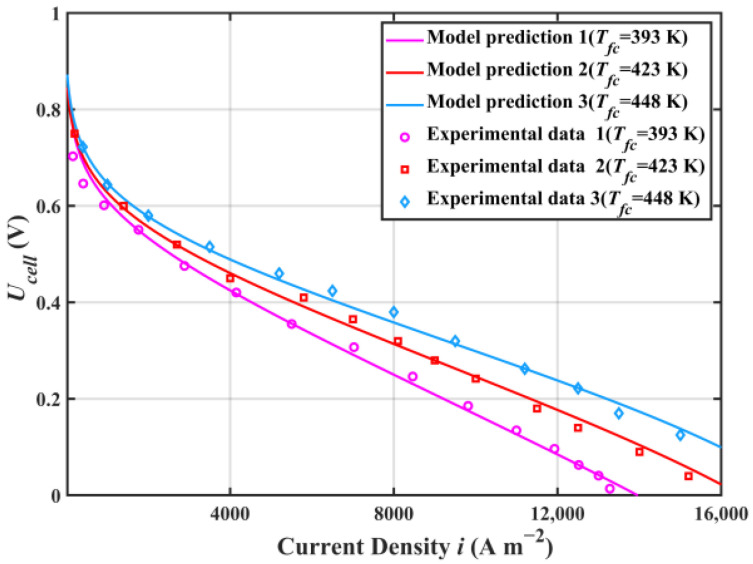
HT-PEMFC single cell voltage model validation (Inlet pressure p=1 atm; The relative humidity of the reaction gas RH=0.38% ).

Figure 1 shows the comparison of the HT-PEMFC single cell voltage and experimental data from [41]. This paper compares the predicted voltage of the HT-PEMFC model with the experimental voltage at the operating temperatures of 393, 423 and 448 K. The results showed that the output voltage was in good agreement with the experimental data. The slight error might be because the model did not consider the effects of factors such as temperature, pressure, and reactant concentration on the limiting current density [42].

### 2.2. Application of 6FPBI Membranes

**Figure 2:**
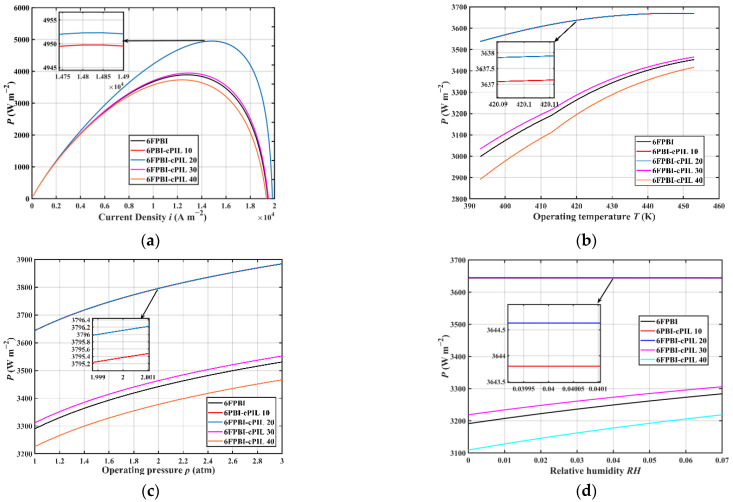
The power density of the HT-PEMFC single cell using 6FPBI membranes under different (**a**) current density, (**b**) operating temperature, (**c**) operating pressure, and (**d**) relative humidity.

Figure 2 presents the power density of the HT-PEMFC single cell using 6FPBI membranes under different operating conditions. The HT-PEMFC single cell applied with the 6FPBI-cPILs 20 membrane had the highest power density. Compared with the HT-PEMFC applied with a 6FPBI membrane, the power density of the cell was higher when the HT-PEMFC used a 6FPBI membrane with cPIL doping weighting ratios of 10%, 20%, and 30%, even at a higher operating temperature, pressure, and relative humidity. Therefore, the addition of appropriate cPIL to the 6FPBI membrane was beneficial to improving the power density of the HT-PEMFC. Further increases in cPIL content led to a more compact chemical structure of the membrane, which resulted in a lower PA doping ability and proton conductivity. Figure 2a shows that the power density of the HT-PEMFC single cell reached up to 4952.3 $W\cdot m^{-2}$ when 6FPBI-cPIL 20 membranes were applied. According to Figure 2b,c, the power density of the HT-PEMFC single cell increased with an increasing operating temperature and pressure. In addition, Figure 2d indicates that the increase in relative humidity was beneficial to the increase in proton conductivity of the membrane, thus improving the power density of the HT-PEMFC.

### 2.3. Parametric Studies

According to the analysis of membrane applications, the 6FPBI-cPIL 20 membrane performed better in the HT-PEMFC, while the application of the 6FPBI-cPIL 40 membranes reduced the HT-PEMFC performance. Therefore, the 6FPBI, 6FPBI-cPIL 20, and 6FPBI-cPIL 40 membranes were chosen to apply to the system for studying its performance.

#### 2.3.1. Effect of Current Density

**Figure 3:**
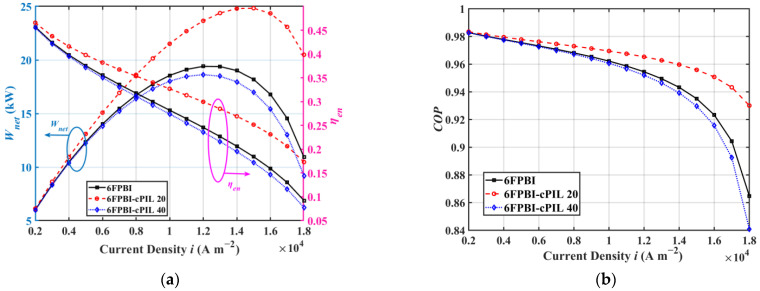
Variation of HT-PEMFC system performance with current density: (**a**) output power $W_{net}$ and energy efficiency $\eta_{en}$; (**b**) the coefficient of performance COP.

Figure 3 presents the variations of HT-PEMFC system performances with current densities. Figure 3a shows that the HT-PEMFC system applied with the 6FPBI-cPIL 20 membranes reached 24.83 kW. Compared with the HT-PEMFC system with undoped 6FPBI membranes, the use of 6FPBI-cPIL 20 membranes increased the net output power of the HT-PEMFC system by 5.43 kW. The system output power can be maximized in the higher current density region, but the energy efficiency would be lower. For stationary applications, the current density can be reduced to improve energy efficiency when sufficient installation space is available. However, for vehicles with limited installation space, a high current density operating mode is necessary to achieve high power density in the HT-PEMFC system. Therefore, the appropriate current density should be selected according to the requirements of the power system in practical applications. According to Figure 3b, the use of appropriately weighted ionic liquids could effectively improve the performance factor of the system. This is mainly due to the significantly enhanced phosphate doping ability of the 6FPBI-cPIL membranes after doping with cPIL, which resulted in a high proton conductivity. In addition, the coefficient of performance (COP) for the HT-PEMFC system decreased as the current density increased, which was mainly due to the higher current density increasing the power consumption of the auxiliary devices.

#### 2.3.2. Effect of Inlet Temperature

**Figure 4:**
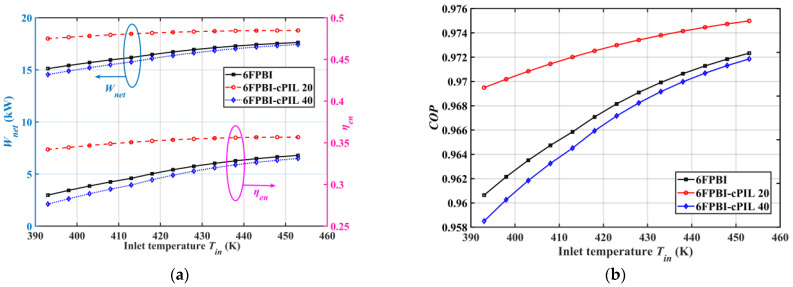
Variation of system performance with inlet temperature: (**a**) output power $W_{net}$ and energy efficiency $\eta_{en}$; (**b**) the coefficient of performance COP.

Figure 4 illustrates the effect of inlet temperature on system performance. According to Figure 4a, the increase in temperature was beneficial to improving the net output power and efficiency of the system. The higher temperatures effectively enhanced Brownian motion and accelerated proton transfer, leading to an overall increase in proton conductivity. In PA-doped membranes, proton conduction depends mainly on the conduction of phosphate molecules and the interaction of the polymer with phosphate [43]. Figure 4b shows that the increase in temperature enhanced the system COP. The output performance of the HT-PEMFC applied with the 6FPBI-cPIL 20 membrane was better in the studied operating temperature variation range. Compared with non-doped cPIL membranes, the doping of ionic liquid compounds at higher temperatures also led to higher proton conductivity to enhance HT-PEMFC system performance.

#### 2.3.3. Effect of Inlet Hydrogen Pressure

**Figure 5:**
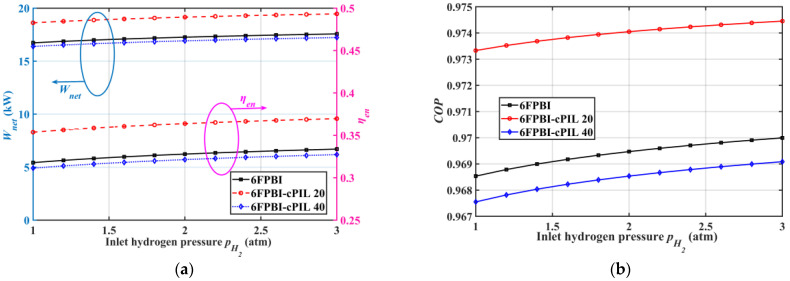
Variation of system performance with inlet hydrogen pressure: (**a**) output power $W_{net}$ and energy efficiency $\eta_{en}$; (**b**) the coefficient of performance COP.

Figure 5 shows the effect of inlet hydrogen pressure on the performance of HT-PEMFC systems using different membranes. Figure 5a shows that the power generation and energy efficiency of the HT-PEMFC system increased slightly with increasing inlet hydrogen pressure. This is because the increase in hydrogen pressure helped the stack to produce more power, and the increase in hydrogen pressure did not require power consumption in the studied HT-PEMFC system. Figure 5b shows that the COP of the HT-PEMFC system using 6FPBI-cPIL 20 membranes improved by only 0.41% when the stack inlet hydrogen pressure rose from 1 to 3 atm. Although the influence of hydrogen pressure on system performance was relatively less, an increase in inlet hydrogen pressure could slightly improve the performance of the system regardless of the type of membrane used.

#### 2.3.4. Effect of Inlet Oxygen Pressure

**Figure 6:**
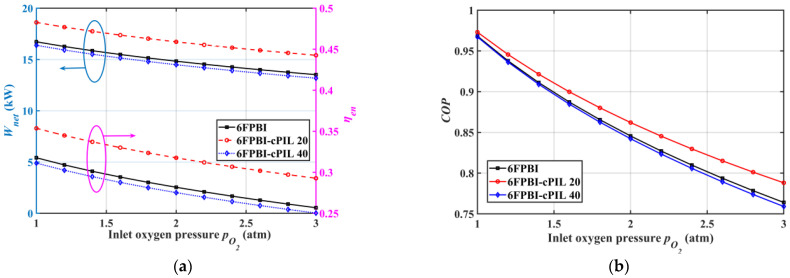
Variation of system performance with inlet oxygen pressure: (**a**) output power $W_{net}$ and energy efficiency $\eta_{en}$; (**b**) the coefficient of performance COP.

The effect of inlet oxygen pressure on the performance of HT-PEMFC systems using different membranes is presented in Figure 6. According to Figure 6a, the increase in oxygen pressure decreased the output power and efficiency of the system. Although the increased pressure was helpful to improve the HT-PEMFC stack power density, it required the air compressor to consume a lot of power. Figure 6b shows that the system COP with the 6FPBI-cPIL 20 membranes was still higher than that of the system without doped ionic liquid at a different oxygen pressure. Furthermore, the performance of the HT-PEMFC system decreased with increasing oxygen pressure. Therefore, the system cathode pressure should be kept as low as possible to reduce power consumption and membrane oxidation for better system output performance.

## 3. Methods and Materials

### 3.1. Preparation and Properties of 6FPBI-cPIL Membranes

#### 3.1.1. 6FPBI-cPIL Membranes Preparation

**Figure 7:**
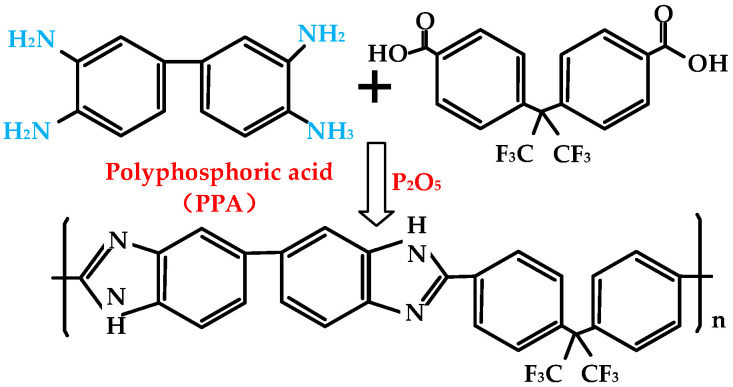
Synthesis process of 6FPBI.

**Figure 8:**
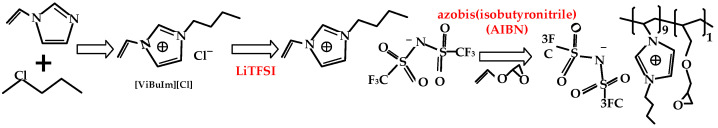
Synthesis process of cPIL.

Figure 7 shows the synthesis process of 6FPBI. 6FPBI was synthesized by a typical polycondensation reaction. The preparation materials of 6FPBI mainly included: 3,3′-Diaminobenzidine (DAB), 2,2-Bis(4-carboxyphenyl) hexafluoro propane, polyphosphoric acid (PPA), and P_2_O_5_. The details of the preparation and the synthetic procedure can be referred to in the previous studies [32,44]. Figure 8 presents the synthesis process of cPIL. The preparation materials of cPIL mainly included: 1-chlorobutane, 1-vinylimidazol, lithium bis(trifluoromethanesulfonyl)imide (LiTFSI), and azobis(isobutyronitrile) (AIBN). The synthesis of cPILs consisted of three main steps: the synthesis of double-bonded ionic liquid 1-vinyl-3-butylimidazolium chloride ([ViBuIm]Cl), replacement of [ViBuIm]Cl by anion exchange reaction, and copolymerization of the obtained double-bonded ionic liquid with the crosslinker. The specific preparation process of the 6FPBI-cPIL membrane can be found in [32]. The 6FPBI-cPIL composite membranes were prepared with a weight ratio of 10%, 20%, 30%, and 40% in the obtained composite membranes and are named 6FPBI-cPIL 10, 6FPBI-cPIL 20, 6FPBI-cPIL 30, and 6FPBI-cPIL 40, respectively [31]. Liu et al. [32] dissolved [ViBuIm]TFSI in dimethyl sulfoxide-d6 (DMSO-d6) and characterized it with ^1^H NMR spectroscopy. The results revealed that the chemical shifts of 7.93, 8.18, and 9.50 ppm were attributed to the attached imidazolium cations, which proved that [ViBuIm]TFSI was successfully prepared. The Fourier transform infrared spectra (FTIR) of cPIL, 6FPBI, and all composite membranes can be found in [32]. In addition, all membranes prepared had a good thermal stability below 200 °C and were suitable for application in HT-PEMFCs [32].

#### 3.1.2. 6FPBI-cPIL Membranes Performance

The electrical conductivity of PA-PBI membranes depended on the amount of phosphoric acid doped in the polymer. The dried membrane samples were immersed in 80 wt % H_3_PO_4_ at 80 °C for 24 h. The PA doping level (DL) of the membrane was calculated with [32]:

(1) $$DL=\frac{(w_{doped}-w_{doped})/M_{PA}}{(w_{undoped}\times \left(1-X\%\right))/M_{PBI}}$$

where $w_{doped}$ and $w_{doped}$ are the weights of the membrane samples before and after immersion, respectively. X% is the weight percentage of cPIL in the membrane. $M_{PA}$ and $M_{PBI}$ represent the molecular weight of PA and repeat unit of 6FPBI, respectively. The measurement of proton conductivity of PA-PBI membranes was conducted with the four-electrode AC impedance method by Liu et al. [32]. We developed a fuel cell system model to predict the performance of the HT-PEMFC using the 6FPBI membrane based on the test results.

**Figure 9:**
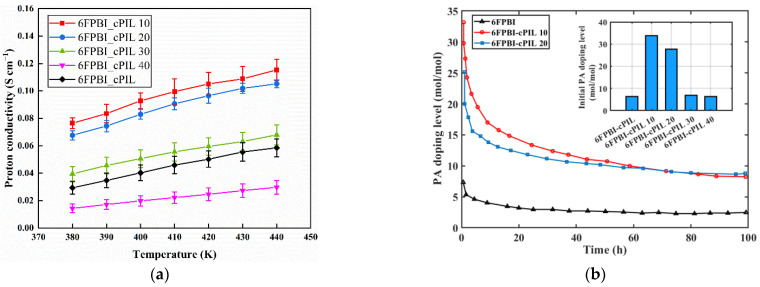
Performance of different membranes: (**a**) variation of proton conductivity with temperature; (**b**) variation of PA doping level with time.

Figure 9 illustrates the performance of different membranes. Figure 9a shows that 6FPBI-cPIL 10 and 6FPBI-cPIL 20 had the highest proton conductivity. Since the 6FPBI-cPIL 10 membrane displayed a poor mechanical property after doping with phosphoric acid [32], the performance was better when the weight ratio was 20% of the cPIL. In addition, the proton conductivity of all membranes increased with increasing temperature. The proton conductivity of the membrane depended on the concentration of phosphoric acid. According to Figure 9b, the PA doping level of all membranes gradually decreased with increasing time. When the test time reached 96 h, the PA doping level of 6FPBI-cPIL 20 membranes was higher than that of 6FPBI-cPIL 10. Compared with 6FPBI membranes, the appropriate cPIL doping could effectively improve phosphate retention and the long-term stability of the proton conductivity of the membrane.

### 3.2. Thermodynamic Modeling

#### 3.2.1. HT-PEMFC Single Cell Model

**Figure 10:**
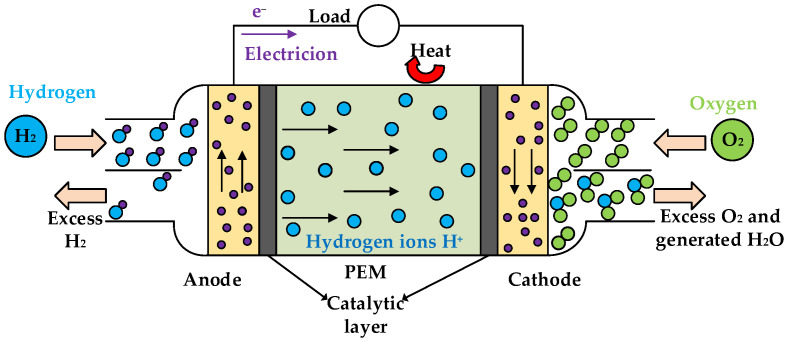
Reaction mechanism of HT-PEMFCs.

Figure 10 illustrates the reaction mechanism of HT-PEMFCs. HT-PEMFCs mainly consist of a cathode, anode, proton exchange membrane (PEM) and catalytic layer. HT-PEMFCs directly convert the chemical energy of hydrogen and oxygen into electrical and thermal energy. The electrochemical reactions at the anode and cathode of HT-PEMFCs are $H_{2}\to 2H^{+}+2e^{-}$ and $2H^{+}+\frac{1}{2}O_{2}+2e^{-}\to H_{2}O+\mathrm{heat}$, respectively. The total electrochemical reaction is described as: $H_{2}\left(g\right)+\frac{1}{2}O_{2}\left(g\right)\to H_{2}O\left(g\right)+\mathrm{heat}+\mathrm{electricity}$. The generated electrical power can be used for external loads. For HT-PEMFCs using PA-PBI membranes, the mechanism of proton conduction in the membranes is known as the “Grotthuss mechanism”. The electrochemical reaction mechanism can be described as [45]:

(2) $$\mathrm{Anode}:H_{2}{\mathrm{PO}}_{4}^{-}+H^{+}=H_{3}{\mathrm{PO}}_{4}$$

(3) $$\mathrm{Cathode}:\;\mathrm{PBI}\cdot H^{+}=\mathrm{PBI}+H^{+}$$

(4) $$\mathrm{Membrane}:H_{3}{\mathrm{PO}}_{4}+\mathrm{PBI}=H_{2}{\mathrm{PO}}_{4}^{-}+\mathrm{PBI}\cdot H^{+}$$

When the external electrical load does not require current, the HT-PEMFC reaches its theoretical maximum voltage, also known as the reversible voltage $E_{r}$. The reversible output voltage $E_{r}$ can be obtained by [37]:

(5) $$E_{r}=E_{r}^{0}+\frac{\Delta S}{nF}\left(T-T_{0}\right)+\frac{RT}{nF}ln(\frac{p_{H_{2}}p_{O_{2}}^{0.5}}{p_{H_{2}O}})$$

where $E_{r}^{0}=1.185\;V$ is the ideal standard potential [37]; ΔS is the standard molar enthalpy; n=2 is the number of electrons exchanged per hydrogen molecule; F is the Faraday constant; T is the operating temperature; R is a gas constant; $p_{H_{2}}$, $p_{O_{2}}$, and $p_{H_{2}O}$ are partial pressures of hydrogen, oxygen, and water vapor, respectively.

When the HT-PEMFC generates current, three types of overpotentials are generated: activation, concentration, and ohmic overpotentials. The activation overpotential $E_{act}$ is [37]:

(6) $$E_{act}=\frac{RT}{\alpha nF}ln(\frac{i+i_{leak}}{i_{0}})$$

where α is the charge transfer coefficient; i, $i_{leak}$, and $i_{0}$ are the operating, leak, and exchange current densities, respectively.

The concentration overpotential $E_{con}$ is [46]:

(7) $$E_{con}=\left(1+\frac{1}{\alpha }\right)\frac{RT}{nF}\ln(\frac{i_{L}}{i_{L}-i-i_{leak}})$$

where $i_{L}$ is the limiting current density.

The concentration overpotential $E_{ohm}$ is [47]:

(8) $$E_{ohm}=i\left(\frac{t_{mem}}{\sigma_{mem}}\right)$$

where $t_{mem}$ is the thickness of PEM. $\sigma_{mem}=\frac{A_{0}B}{T}e^{\frac{-c_{act}}{RT}}$ is the proton conductivity of the PEM electrolyte. $A_{0}$, B, and $c_{act}$ are obtained by fitting from experimental data [48]:

(9) $$A=68DL^{3}-6324DL^{2}+65750DL+8460$$

(10) $$B=\{\begin{array}{c} 1+\left(0.01704T-4.767\right)RH\;373.15K\leq T\leq 413.15\\ 1+\left(0.1432T-56.89\right)RH\;\;413.15K<T\leq 453.15\\ 1+\left(0.7T-309.2\right)RH\;\;\;\;\;\;\;\;\;453.15<T\leq 473.15 \end{array}$$

(11) $$c_{act}=-619.6DL+21750$$

where RH is the relative humidity of the electrolyte. DL is the PA doping level, depending on the phosphoric acid concentration, doping temperature, and soaking time. The conductivity of the PA-PBI polymer depends on the amount of phosphoric acid doped in the polymer.

The voltage of a HT-PEMFC single cell can be obtained by [49]:

(12) $$U_{cell}=E_{rev}-E_{act}-E_{ohm}-E_{con}$$

The power output P of HT-PEMFC can be calculated by [50]:

(13) $$P=iAU_{cell}$$

#### 3.2.2. System Description

**Figure 11:**
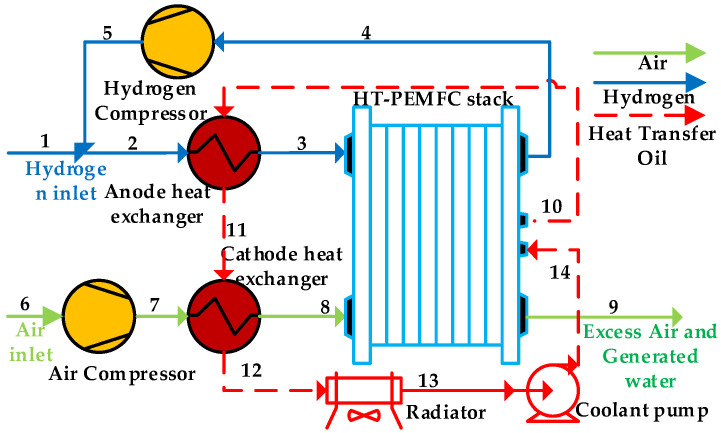
Schematic illustration of the HT-PEMFC system.

Figure 11 shows the schematic illustration of the HT-PEMFC system. It is suitable to be used for vehicle, portable, and stationary applications. Hydrogen from the hydrogen storage tank is regulated to the operating pressure of the stack and then enters the system. The fresh hydrogen is mixed with the hydrogen recovered from the hydrogen compressor (HC). The HC can recover unreacted hydrogen from the stack to improve fuel utilization. The mixed hydrogen is preheated to the operating temperature in the anode heat exchanger (AHE) and then enters the stack. Air from the environment is pressurized and heated to an operating pressure and temperature by an air compressor (AC) and cathode heat exchanger (CHE) before entering the stack. Then, the excess air and generated water are exhausted at the cathode outlet of the stack. The heat generated by the HT-PEMFC stack is carried away by the heat transfer oil and preheats the reaction gas at the inlet of the stack. The excess heat is released into the environment through the radiator (R).

#### 3.2.3. System Model

A zero-dimensional PEMFC model was established in this study. To simplify the calculation and make the parameter analysis reasonable, the following reasonable assumptions were made:

- The system works under steady-state conditions;
- All gases are ideal and ignore the pressure drop in the pipeline;
- The temperature rise of the coolant and the two reactants in the stack are fixed at 5 K and the pressure drop is fixed at 0.2 atm;
- The relative humidity of the reaction gas *RH* = 7.6%;
- The performance of the HT-PEMFC stack remains consistent with the single cell.

The output power $W_{stack}$ and the generated heat $Q_{stack}$ of the HT-PEMFC stack were given by [48]:

(14) $$W_{stack}=N_{cell}\cdot U_{cell}\cdot i\cdot A$$

(15) $$Q_{stack}=N_{cell}\cdot \left(E_{rev}-U_{cell}\right)\cdot i\cdot A$$

The air compressor power consumption $W_{AC}$ and the hydrogen compressor power consumption $W_{HC}$ were calculated by [51]:

(16) $$W_{AC}=\frac{C_{p,6}{\dot{m}}_{6}T_{6}}{\eta_{C}}\left({\left(\frac{p_{7}}{p_{6}}\right)}^{\frac{\gamma -1}{\gamma }}-1\right)$$

(17) $$W_{HC}=\frac{C_{p,4}{\dot{m}}_{4}T_{4}}{\eta_{C}}\left({\left(\frac{p_{5}}{p_{4}}\right)}^{\frac{\gamma -1}{\gamma }}-1\right)$$

where $C_{p}$ is the specific heat at constant pressure. $\dot{m}$ is the mass flow rate. γ is the adiabatic coefficient. $\eta_{c}$ is the compressor isentropic efficiency. The numbers in the subscripts represent the working fluid state at different temperatures and pressures. The numbers correspond to the specific fluids in the HT-PEMFC system schematic.

The heat exchange process in the heat exchanger followed the energy conservation [48]:

(18) $${\dot{m}}_{9}C_{p,9}\left(T_{9}-T_{11}\right)={\dot{m}}_{7}C_{p,7}\left(T_{8}-T_{7}\right)+{\dot{m}}_{2}C_{p,2}\left(T_{3}-T_{2}\right)$$

The power consumption of the pump $W_{pump}$ was [51]:

(19) $$W_{pump}=\frac{{\dot{m}}_{13}\left(p_{14}-p_{13}\right)}{\rho \eta_{pump}}$$

The net output power $W_{net}$ and energy efficiency $\eta_{en}$ of the system were obtained by [51]:

(20) $$W_{net}=W_{stack}-W_{AC}-W_{HC}-W_{pump}$$

(21) $$\eta_{en}=\frac{W_{net}}{{\dot{m}}_{1}\cdot LHV_{H_{2}}}$$

The coefficient of performance (COP) for the HT-PEMFC system is defined as the ratio of the total output energy to the total generated energy [52]:

(22) $$COP=\frac{W_{net}}{W_{stack}}$$

## 4. Conclusions

The performance of the HT-PEMFC system using the 6FPBI membrane doped with cPIL as the electrolyte was investigated in this study. The HT-PEMFC single cell model was developed and validated considering the degree of phosphate doping of the 6FPBI composite membrane. The power density of the HT-PEMFC single cell reached up to 4952.3 $W\cdot m^{-2}$ when 6FPBI-cILs 20 membranes were applied. The simulation results suggested that the 6FPBI membrane doping-appropriate cPIL was beneficial to improve the HT-PEMFC output performance, while excessive cPIL content led to performance degradation.

The performance and parameter studies of HT-PEMFC systems using different ionic membranes were conducted. The results suggested that the current density should be as low as possible to improve the system energy efficiency when the HT-PEMFC system output power meets the requirements. The HT-PEMFC system using the 6FPBI-cPIL 20 membrane also showed higher performance, even at higher temperatures and pressures, than the systems using 6FPBI membranes. In addition, the HT-PEMFC system should be operated at a high inlet temperature and hydrogen pressure to increase system output power and efficiency. The oxygen inlet pressure should be reduced to decrease the power consumption of the ancillary equipment. The proposed model serves as a prediction for the performance of HT-PEMFCs with the application of advanced ionic membranes. The study of membrane application and operating parameters provides a reference for the future design and optimization of HT-PEMFC systems.

## Figures and Tables

**Table 1:** Input parameters for system thermodynamic modeling.

Components	Parameters	Values
HT-PEMFC stack	Number of fuel cells, $N_{cell}$	175
	Effective working area, A	0.03 m^2^ [38]
	Anode stoichiometry, $\lambda_{H_{2}}$	1.05 [39]
	Cathode stoichiometry, $\lambda_{O_{2}}$	2.0 [39]
	Anode inlet pressure, $p_{H_{2}}$	2 atm [39]
	Cathode inlet pressure, $p_{O_{2}}$	2 atm [39]
	Current density, i	$8000\;A\cdot m^{-2}$ [37]
	Inlet temperature, $T_{in}$	423 K [37]
Compressors	Isothermal efficiency, $\eta_{C}$	80% [40]
Pump	Isothermal efficiency, $\eta_{pump}$	80% [40]
Heat exchangers	Pinch point temperature difference	10 K [40]

## Data Availability

Not applicable.
